# Xeniaphyllane-Derived Terpenoids from Soft Coral *Sinularia nanolobata*

**DOI:** 10.3390/md16020040

**Published:** 2018-01-24

**Authors:** Fu-Yun Hsu, Shang-Kwei Wang, Chang-Yih Duh

**Affiliations:** 1 Department of Marine Biotechnology and Resources, National Sun Yat-sen University, Kaohsiung 804, Taiwan; m00502034@student.nsysu.edu.tw; 2 Department of Microbiology and Immunology, Kaohsiung Medical University, Kaohsiung 807, Taiwan

**Keywords:** *Sinularia nanolobata*, tetranorditerpenoid, norditerpenoid, gibberosin J, cytotoxicity

## Abstract

A novel tetranorditerpenoid, sinubatin A (**1**) (having an unprecedented carbon skeleton), a new norditerpenoid, sinubatin B (**2**) (a 4,5-epoxycaryophyllene possessing an unusual methylfuran moiety side chain), and a known diterpenoid, gibberosin J (**3**) were isolated from soft coral *Sinularia* *nanolobata.* The structures of the new compounds were elucidated by extensive analysis of spectroscopic data.

## 1. Introduction

**Figure 1:**
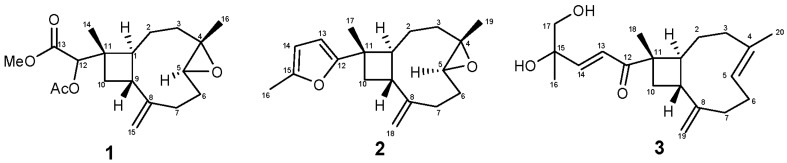
Structure of Metabolites **1**–**3**.

Soft corals of genus *Sinularia* (*Alcyoniidae*) have been reported to be a rich source of novel structures and bioactive terpenoids and steroids [1]. Previous studies on the sample of *Sinularia nanolobata* Verseveldt have resulted in the isolation of diterpenoids [2,3,4,5] and sesquiterpenoids [3,4], and steroids [5,6]. During the course of our search of bioactive compounds from marine organisms, a chemical investigation on the secondary metabolites of *S. nanolobata* from Taiwanese waters has afforded a novel tetranorditerpenoid, sinubatin A (**1**) (possessing an unprecedented carbon skeleton), a new norditerpenoid, sinubatin B (**2**) (a 4,5-epoxycaryophyllene possessing an unusual methylfuran moiety side chain), and gibberosin J (**3**) (Figure 1). The structures of **1** and **2** were determined by extensive spectroscopic analysis. The chemical structure of gibberosin J (**3**) was determined by comparison of its infrared (IR), high resolution electron spray ionization mass spectrum (HR-ESI-MS), and nuclear magnetic resonance (NMR) spectroscopic data with the literature data [7].

## 2. Results and Discussion

**Figure 2:**
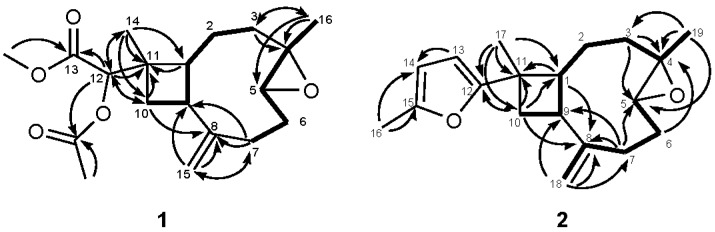
Selected ^1^H–^1^H COSY (bold lines) and HMBC (arrows) correlations of **1** and **2**.

**Figure 3:**
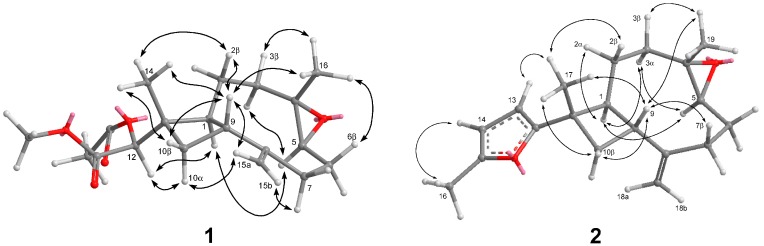
Key NOESY Correlations of **1** and **2**.

Chromatographic separation on the acetone extract resulted in the isolation of two new terpenoids, sinubatin A and B (**1** and **2**), as well as a known compound, gibberosin J (**3**). The HR-ESI-MS, ^13^C NMR, and DEPT spectroscopic data of sinubatin A (**1**) established its molecular formula as C_19_H_28_O_5_. ^13^C NMR and DEPT spectrum of **1** showed the presence of four methyl, five sp^3^ methylene, four sp^3^ methine, one sp^2^ methylene, two sp^3^ quaternary, one sp^2^ quaternary, and two carbonyl carbons. The presence of an exomethylene in **1** was shown by the NMR data [δ_H_ 4.90 (1H, s), 5.01 (1H, s); δ_C_ 114.0 (CH_2_), 150.7 (C)] (Table 1). The NMR data [δ_C_ 59.6 (C), 63.8 (CH), δ_H_ 2.92 (1H, dd, *J* = 10.8, 4.0 Hz)] (Table 1) indicated a trisubstituted epoxide in **1**. The NMR spectrum contained signals for a secondary acetoxyl [δ_H_ 4.74 (1H, s), 2.15 (3H, s); δ_C_ 79.4 (CH), 20.6 (CH_3_), and 170.8 (C)] (Table 1). The presence of a methyl ester [δ_H_ 3.71 (3H, s); δ_C_ 51.9 (CH_3_), 169.2 (C)] was shown in the NMR spectrum. From the data of ^1^H–^1^H COSY correlations (Figure 2), we established two partial structures of consecutive proton systems extending from H-10 to H-3 through H-9 and from H-16 to H-7 through H-4. HMBC correlations of (a) CH_3_-16 to C-3, C-4, and C-5, (b) H_2_-15 to C-7, C-8, and C-9, (c) CH_3_-14 to C-1, C-10, C-11, and C-12, (d) CH-12 to C-10, C-11,C-13, and C-14 connected four partial structures and concluded the planar structure of **1**, as shown in Figure 2. The above functionalities revealed that sinubatin A (**1**) possesses a novel xeniaphyllane-derived tetranorditerpene skeleton. The relative configuration of **1** was established from a NOESY experiment. NOE correlations of H_3_-14/H-9 and H_3_-16/H-9 pointed H_3_-14, H-9 and H_3_-16 to be on the β-side of the molecule. NOE correlation of H-1/H-5 suggested H-1 and H-5 were on the α-side of the molecule. (Figure 3).

HR-ESI-MS of sinubatin B (**2**) showed a pseudomolecular ion peak at *m*/*z* 309.1842 [M + Na]^+^, consistent with the molecular formula C_19_H_2__6_O_2_, and seven degrees of unsaturation. The ^13^C NMR spectrum (Table 2) of **2** displayed 19 carbon signals, and a DEPT experiments indicated the presence of three methyl, five sp^3^ methylene, three sp^3^ methine, two sp^2^ methine, one sp^2^ methylene, two sp^3^ quaternary, and three sp^2^ quaternary carbons. The ^13^C and ^1^H NMR spectra (Table 2) revealed the presence of a trisubstituted epoxides [δ_H_ 2.92 (dd, *J* = 10.4, 4.0 Hz); δ_C_ 63.7 (CH) and 59.7 (C)], a 2,5-disubstituted furan [δ_H_ 5.83 (d, *J* = 4.0 Hz), 5.84 (dd, *J* = 4.0, 0.8 Hz), and 2.28 (d, *J* = 0.8 Hz); δ_C_ 103.7 (CH), 105.8 (CH), 150.6 (C), 160.2 (C), 13.6 (CH_3_)] [8] and an exomethylene [δ_H_ 5.09 (s) and 4.93 (s); δ_C_ 113.5 (CH_2_) and 151.4 (C)]. Thus, the tetracyclic structure of **2** was revealed. From the ^1^H–^1^H COSY spectrum of **2**, it was also possible to identify two different structural units (Figure 2), which were assembled with the assistance of an HMBC experiments. Key HMBC correlations (Figure 2) of H_3_-19 to C-3, C-4, and C-5; H_3_-18 to C-7, C-8, and C-9; H_3_-17 to C-1, C-10, C-11, and C-12 indicated that compound **2** was a 4,5-epoxycaryophyllene having a methylfuran on C-11. The relative configuration of **2** was determined from a NOESY experiment. NOE correlations of H_3_-19/H-9 and H_3_-17/H-9 suggested H_3_-19, H-9 and H_3_-17 to be on the β-side of the molecule. NOE correlation of H-1/H-5 indicated H-1 and H-5 were on the α-side of the molecule. (Figure 3). Compound **2** was the first caryophyllene possessing a methylfuran on C-11.

Compounds **1**–**3** were tested for cytotoxicity against mouse lymphocytic leukemia (P-388), human colon adenocarcinoma (HT-29), and human lung epithelial carcinoma (A-549) tumor cell lines. Compound **3** exhibited cytotoxicity against P-388, A549, and HT-29 with ED_50_ values of of 1.0, 1.2, and 0.5 μg/mL, respectively. However, compounds **1** and **2** were not cytotoxic to P-388, A549 and HT-29 cell lines. Compounds **1**–**3** were also examined for antiviral activity against human cytomegalovirus (HCMV) and did not show anti-HCMV activity.

## 3. Experimental Section

### 3.1. General Experimental Procedures

Optical rotations were obtained on a JASCO P1020 digital polarimeter (Tokyo, Japan). UV and IR spectra were determined on JASCO V-650 (JASCO, Tokyo, Japan) and JASCO FT/IR-4100 spectrophotometers (JASCO, Tokyo, Japan), respectively. NMR spectra were recorded on a Varian MR 400 NMR spectrometer (Santa Clara, CA, USA) at 400 MHz for ^1^H and 100 MHz for ^13^C. ^1^H NMR chemical shifts are expressed in δ (ppm) referring to the solvent peak δ_H_ 7.27 for CHCl_3_ and coupling constants are expressed in Hertz (Hz). ^13^C NMR chemical shifts are expressed in δ (ppm) referring to the solvent peak δ_C_ 77.0 for CDCl_3_. MS were obtained by a Bruker APEX II mass spectrometer (Bruker, Bremen, Germany). Precoated silica gel plates (Merck, Kieselgel 60 F_254_, 0.25 mm) and precoated RP-18 F_254s_ plates (Merck) were used for thin-layer chromatography (TLC) analysis. Silica gel 60 (Merck, Darmstadt, Germany, 230–400 mesh) and LiChroprep RP-18 (Merck, 40–63 µm) were used for column chromatography. High-performance liquid chromatography (HPLC) (Hitachi, Tokyo, Japan) was carried out using a Hitachi L-7100 pump (Hitachi, Tokyo, Japan) equipped with a Hitachi, L-7400 UV detector (Hitachi, Tokyo, Japan) at 220 nm together with a semi-preparative reversed-phased column (Merck, Hibar LiChrospher RP-18e, 5 µm, 250 mm × 25 mm).

### 3.2. Animal Material

The soft coral *S. nanolobata* was collected by hand using scuba at San-Shin-Tai, Taitong County, Taiwan, in July 2008, at a depth of 7 m. A voucher specimen (SST-009) was deposited in the Department of Marine Biotechnology and Resources, National Sun Yat-sen University.

### 3.3. Extraction and Separation

The frozen soft coral (3.0 kg) was chopped into small pieces (about 1 cm) and extracted with acetone in a percolator at room temperature. The acetone extract (30.0 g) of *S. nanolobata* was concentrated under reduced pressure to a brown gum, which was partitioned between EtOAc and H_2_O. The EtOAc-soluble fraction (30 g) was applied to Si 60 CC using *n*-hexane–EtOAc mixtures of increasing polarity for elution. Fraction 12, eluted with *n*-hexane–EtOAc (6:1), was further purified by reverse-phase HPLC (MeOH–H_2_O, 60:40) to obtain **1** (1.5 mg). Fraction 3, eluted with *n*-hexane–EtOAc (80:1), was further purified by reverse-phase HPLC (MeOH–H_2_O, 85:15) to afford **2** (2.6 mg). Fraction 18, eluted with *n*-hexane–EtOAc (1:4), was further purified by reverse-phase HPLC (MeOH–H_2_O, 65:35) to obtain **3** (5.0 mg).

Sinubatin A (**1**): Colorless oil; ${\left[\alpha \right]}_{D}^{25}$ −19.2 (*c* 0.38, CHCl_3_); IR (neat) ν_max_ 2934, 1742, 1442, 1373 and 1420 cm^−1^; ^1^H and ^13^C NMR data, see Table 1; ESI-MS *m*/*z* 359 [M + Na]^+^; HR-ESI-MS *m*/*z* 359.1837 (calcd. for C_19_H_28_O_5_Na, 359.1834).

Sinubatin B (**2**): Colorless oil; ${\left[\alpha \right]}_{D}^{25}$ +17.2 (*c* 0.65, CHCl_3_); IR (neat) ν_max_ 2961, 2925, 1261, 1094, 1020, 799 cm^−1^; ^1^H and ^13^C NMR data, see Table 2; ESIMS *m*/*z* 309 [M + Na]^+^; HR-ESI-MS *m*/*z* 309.1832 (calcd. for C_19_H_26_O_2_Na, 309.1830).

### 3.4. Biological Assay

Cytotoxicity assay and anti-HCMV assay were conducted as previously described [9].

## 4. Conclusions

The chemical study of soft coral *S. nanolobata* led to the isolation of a novel tetranorditerpenoid, sinubatin A (**1**) (having an unprecedented carbon skeleton), a new norditerpenoid, sinubatin B (**2**) (a 4,5-epoxycaryophyllene possessing an unusual methylfuran moiety side chain), and gibberosin J (**3**). Compound **3** exhibited cytotoxicity toward P-388, A549, and HT-29 with ED_50_ values of 1.0, 1.2. and 0.5 μg/mL, respectively. However, compounds **1** and **2** were not cytotoxic to P-388, A549 and HT-29 cell lines. Compounds **1**–**3** did not show anti-HCMV activity.

## Figures and Tables

**Table 1:** NMR spectral data of **1**.

Position	δ_H_ *^a^*	(*J* in Hz)	δ_C_ *^b^*	Type	COSY	HMBC	NOESY
1	2.34	m	45.3,	CH	2, 9	11, 12	5
2α	1.45	m	27.7,	CH_2_	1	-	-
2β	1.57	m			1, 3β	-	9
3α	1.00	td (12.8, 4.8)	38.4,	CH_2_	2β	1, 4, 16	3β, 5
3β	2.09	m			2β	-	3α
4	-	-	59.6,	C		-	-
5	2.92	dd (10.8, 4.0)	63.8,	CH	6β, 16	-	1, 3α
6α	2.30	m	30.2,	CH_2_	-	-	-
6β	1.31	m			5	-	6α
7α	2.16	m	29.2,	CH_2_	6α	6, 9	6β
7β	2.32	m			6α	8, 15	-
8	-	-	150.7,	C		-	-
9	2.71	td (9.6, 9.2)	48.7,	CH	1, 10α, 10β	-	2β, 10β, 14, 15a
10α	1.85	dd (10.4, 9.6)	36.2,	CH_2_	9	9, 11, 12, 14	-
10β	1.74	dd (10.4, 9.2)			9	8	9, 14
11	-	-	38.3,	C	-	-	-
12	4.74	s	79.4,	CH	-	10, 11, 13, 14, carbonyl (OAc-12)	1, 10α
13	-	-	169.2,	qC	-	-	-
14	1.14	s	15.2,	CH_3_	-	1, 10, 11, 12	2α, 2β, 9, 10β
15a	5.01	s	114.0,	CH_2_	-	7, 8, 9	9, 10α, 15b
15b	4.90	s			-	7, 9	7α
16	1.19	s	17.1,	CH_3_	5	3, 4, 5	3β, 6β, 9
OAc-12	2.15	s	20.6,	CH_3_	OMe-13	carbonyl (OAc-12)	-
	-	-	170.8,	C	-	-	-
OMe-13	3.71	s	51.9,	CH_3_	OAc-12	13	-

*^a^* Spectrum recorded at 400 MHz in CDCl_3_. *^b^* Spectrum recorded at 100 MHz in CDCl_3_.

**Table 2:** NMR spectral data of **2**.

Position	δ_H_ *^a^*	(*J* in Hz)	δ_C_ *^b^*	Type	COSY	HMBC	NOESY
1	2.47	td (10.0,8.4)	49.3,	CH	2β, 9	3, 8, 9, 11, 17	2α, 3α, 5
2α	1.72	m	27.2,	CH_2_	3α, 3β	1, 3, 11	1, 3α, 3β
2β	1.54	m			1, 3α, 3β	1	3β, 9
3α	0.98	td (13.2, 5.2)	38.8,	CH_2_	2α, 2β, 19	2, 4, 5, 19	1, 2α, 5
3β	2.07	dt (13.2,3.6)			2α, 2β	-	2α, 2β, 19
4	-	-	59.7,	C	-	-	-
5	2.92	dd (10.4, 4.0)	63.7,	CH	6α, 6β	3, 6	1, 3α, 6α
6α	2.28	m	30.1,	CH_2_	5	4, 5, 7	5
6β	1.37	m			5, 7α, 7β	-	7β
7α	2.44	ddd (12.8, 8.0, 4.0)	29.8,	CH_2_	6β	5, 6, 8, 9	18b
7β	2.17	ddd (12.8, 8.0, 4.4)			6α, 6β	5, 6, 8, 9	9
8	-	-	151.4,	C	-	-	-
9	2.74	td (9.6, 8.4)	47.9,	CH	1, 10α, 10β	1, 7, 8, 10	2β, 7β, 10β, 18a, 19
10α	2.33	dd (10.8, 9.6)	37.8,	CH_2_	9, 10β	9, 11, 12, 17	18a
10β	1.87	dd (10.8, 8.4)			9, 10α	1, 16	9, 10α, 17
11	-	-	36.9,	C	-	-	-
12	-	-	160.2,	C	-	-	-
13	5.83	d (4.0)	103.7,	CH	-	-	17
14	5.84	dd (4.0, 0.8)	105.8,	CH	-	-	16
15	-	-	150.6,	C	-	-	-
16	2.28	d (0.8)	13.6,	CH_3_	-	14, 15	14
17	1.35	s	17.9,	CH_3_	-	1, 10, 11, 12	2α, 2β, 9, 13
18a	5.09	s	113.5,	CH_2_	7β	7, 8, 9	9, 10α
18b	4.93	s			7β	7, 8, 9	7β
19	1.23	s	17.0,	CH_3_	3	3, 4, 5	3β, 9

*^a^* Spectrum recorded at 400 MHz in CDCl_3_. *^b^* Spectrum recorded at 100 MHz in CDCl_3_.
